# Systematic Review of Kidney Involvement in Antisynthetase Syndrome

**DOI:** 10.1016/j.ekir.2026.106669

**Published:** 2026-06-16

**Authors:** Qi-Shun Wu, Ling Yang, Xin Lin, Zhang-Li Wu, Zhi-Liang Yu, Ya-Yun Zeng

**Affiliations:** 1 Division of Nephrology, Department of Medicine, The Second Affiliated Hospital of Wannan Medical University, Wuhu, Anhui, China

**Keywords:** antisynthetase syndrome, autoantibodies, interstitial lung disease, kidney disease, membranous nephropathy, rituximab

## Abstract

**Introduction:**

Antisynthetase syndrome (ASyS) is a rare autoimmune disorder characterized by autoantibodies targeting aminoacyl-tRNA synthetases. Kidney involvement has been historically regarded as uncommon and remains poorly characterized.

**Methods:**

We conducted a systematic review using the Preferred Reporting Items for Systematic Reviews and Meta-Analyses 2020 guidelines (PROSPERO: CRD420261282317). MEDLINE, Scopus, Web of Science, and Google Scholar were searched from inception to January 2026. We included adults with biopsy-proven or clinically defined kidney involvement in ASyS. Risk of bias was assessed using Joanna Briggs Institute tools; certainty of evidence was evaluated using the Grading of Recommendations Assessment, Development and Evaluation framework.

**Results:**

Thirty-eight articles comprising 52 patients were included. Kidney disease presented with or following interstitial lung disease (ILD) in 81% of cases. Among 35 biopsied patients, membranous nephropathy (29%), pauci-immune crescentic glomerulonephritis (23%), acute tubular necrosis (17%), and tubulointerstitial nephritis (11%) were identified. Non-Jo1 antibodies predominated in vasculitic phenotypes and were associated with higher peak creatinine than anti-Jo1 (4.7 vs. 2.3 mg/dl; *P* = 0.01). Corticosteroid monotherapy achieved complete recovery in 48% of mild disease, whereas rituximab was effective in 6 of 8 refractory cases. At a median of 18 months follow-up, 13% progressed to chronic kidney disease (CKD) stages 4 and 5.

**Conclusion:**

Kidney involvement in ASyS carries substantial clinical significance. Non-Jo1 serotype may predict worse kidney outcomes, though this association requires confirmation in prospective cohorts with multivariable adjustment. Routine kidney surveillance and early antibody-guided immunosuppression may improve outcomes, pending validation.

## Graphical abstract

**Figure:**
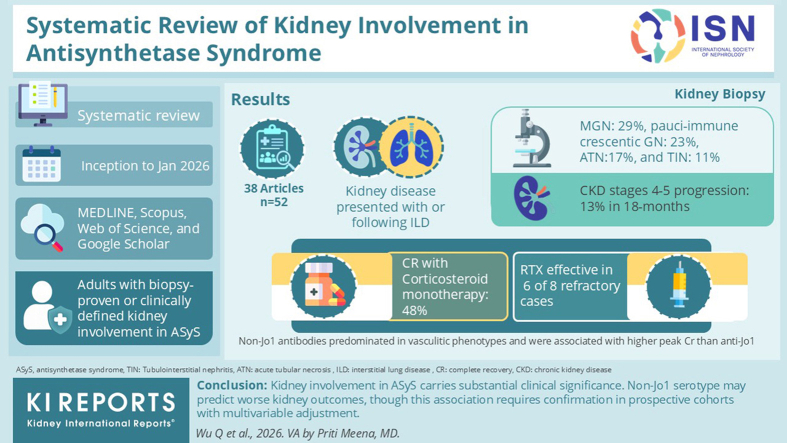

ASyS is an autoimmune disorder characterized by antibodies targeting cytoplasmic aminoacyl-tRNA synthetases.^1^ The classic clinical triad comprises ILD, inflammatory myopathy, and inflammatory arthritis; however, kidney manifestations have been reported sporadically and remain poorly characterized.^2^^,^^3^ Emerging case series have described diverse kidney pathologies, including membranous nephropathy, pauci-immune crescentic glomerulonephritis, and acute tubular necrosis, yet the true incidence, natural history, and optimal therapeutic strategies remain undefined.^4^, ^5^, ^6^

The pathophysiological mechanisms linking aminoacyl-tRNA synthetase autoimmunity to kidney injury remain incompletely elucidated. Shared epitopes between synthetase enzymes and kidney antigens, immune complex deposition, and direct endothelial injury have been proposed.^7^ Furthermore, the prognostic significance of specific autoantibody profiles, particularly the distinction between anti-Jo1 and non-Jo1 serotypes, in kidney involvement remains controversial.^7^

Given these knowledge gaps, we performed a systematic review to characterize the clinical spectrum, histopathological patterns, treatment responses, and long-term kidney outcomes in ASyS-associated kidney disease. Our objective was to synthesize available evidence and provide preliminary recommendations for kidney care in this rare but clinically important condition, while acknowledging the very low certainty of evidence inherent to the source data.

## Methods

This systematic review was conducted using the Preferred Reporting Items for Systematic Reviews and Meta-Analyses 2020 statement.^8^ The protocol was registered with PROSPERO (registration number: CRD420261282317).

### Search Strategy and Selection Criteria

We systematically searched MEDLINE (via PubMed), Scopus, Web of Science, and Google Scholar from inception to January 2026 without language or date restrictions. The search strategy combined medical subject headings (MeSH) and free-text terms: ("kidney involvement" OR "renal involvement" OR "nephropathy" OR "glomerulonephritis" OR "tubulointerstitial nephritis") AND ("antisynthetase syndrome" OR "anti-synthetase syndrome" OR "aminoacyl-tRNA synthetase" OR "anti-Jo1" OR "anti-PL-7" OR "anti-PL-12" OR "anti-EJ" OR "anti-OJ"). The complete search strategy is provided in Supplementary Table S1. Excluded studies with reasons for exclusion are listed in the Supplementary References.

The inclusion criteria were as follows: (i) adult patients (aged ≥ 18 years) with ASyS defined by aminoacyl-tRNA synthetase autoantibody positivity; (ii) biopsy-proven or clinically defined kidney involvement; and (iii) sufficient data on clinical presentation, treatment, or outcomes. The exclusion criteria were as follows: (i) duplicate publications, (ii) reviews without original patient data, (iii) pediatric cases (aged < 18 years), and (iv) insufficient clinical details.

### Definition of Kidney Involvement

Kidney involvement was defined as the presence of ≥ 1 of the following: (i) proteinuria > 0.5 g/24 h or urine protein-to-creatinine ratio > 0.5 g/g, (ii) microscopic hematuria (> 3 red blood cells per high-power field), (iii) elevated serum creatinine (> 1.2 mg/dl or > 106 μmol/l for females, > 1.3 mg/dl or > 115 μmol/l for males) or estimated glomerular filtration rate < 60 ml/min per 1.73 m^2^, and (iv) biopsy-proven kidney pathology. Patients with isolated electrolyte abnormalities without structural kidney damage were excluded.

Two reviewers (Q-SW and LY) independently screened titles, abstracts, and full texts using Covidence systematic review software. Discrepancies were resolved by consensus or consultation with a third reviewer (Z-LY).

### Data Extraction and Quality Appraisal

A standardized data extraction form captured demographic characteristics, autoantibody profiles (including detection methodology where reported), extrarenal manifestations, kidney presentation, histopathological findings, therapeutic regimens, and kidney outcomes. The primary outcome was complete kidney recovery, defined as return to baseline serum creatinine (for known baseline) or normalization of serum creatinine (< 1.2 mg/dl), plus resolution of proteinuria (< 0.3 g/24 h) at 6 months or last follow-up. Secondary outcomes included partial recovery (≥ 50% reduction in proteinuria or ≥ 25% improvement in estimated glomerular filtration rate without reaching complete recovery criteria), progression to CKD (estimated glomerular filtration rate < 60 ml/min per 1.73 m^2^ for ≥3 months), progression to CKD stages 4 and 5 (estimated glomerular filtration rate < 30 ml/min per 1.73 m^2^), and requirement for kidney replacement therapy.

Risk of bias was assessed using the Joanna Briggs Institute critical appraisal tools for case series and case reports.^9^ Certainty of evidence was evaluated using the Grading of Recommendations Assessment, Development and Evaluation framework, adapted for systematic reviews of observational studies.^10^ Risk of bias and the Grading of Recommendations Assessment, Development and Evaluation evidence profiles are presented in Supplementary Figures S1 and Figure S2, respectively. The detailed data extraction form is provided in the Supplementary Methods.

### Data Synthesis

Due to substantial clinical heterogeneity and the predominance of case reports, quantitative meta-analysis was not feasible. We employed narrative synthesis with subgroup analyses according to histopathological pattern and autoantibody status. Continuous variables were expressed as mean ± SD or median (interquartile range), and categorical variables as frequencies and percentages. Between-group comparisons were performed using *t* test or Fisher exact test, where appropriate, with statistical significance set at *P* < 0.05. Given the small sample size (*n* = 52) and retrospective nature of data, multivariable regression analysis was not feasible; therefore, all comparisons should be considered exploratory and hypothesis-generating only, with the acknowledged limitation that observed differences may reflect confounding by age, time to kidney involvement, and other baseline characteristics rather than true group effects.

### Sensitivity Analyses

To assess the robustness of our findings, we conducted the following 3 sensitivity analyses: (i) excluding studies with Joanna Briggs Institute quality scores < 6 (low-quality case reports), (ii) restricting to biopsy-proven cases only (excluding clinically defined kidney involvement), and (iii) excluding studies published before 2010 to account for evolving diagnostic criteria. We compared the distribution of histopathological patterns and treatment response rates between the full cohort and each restricted cohort. Results were considered robust if the direction and magnitude of associations remained consistent across analyses. Complete sensitivity analysis results are provided in Supplementary Table S2.

## Results

### Literature Flow

**Figure 1:**
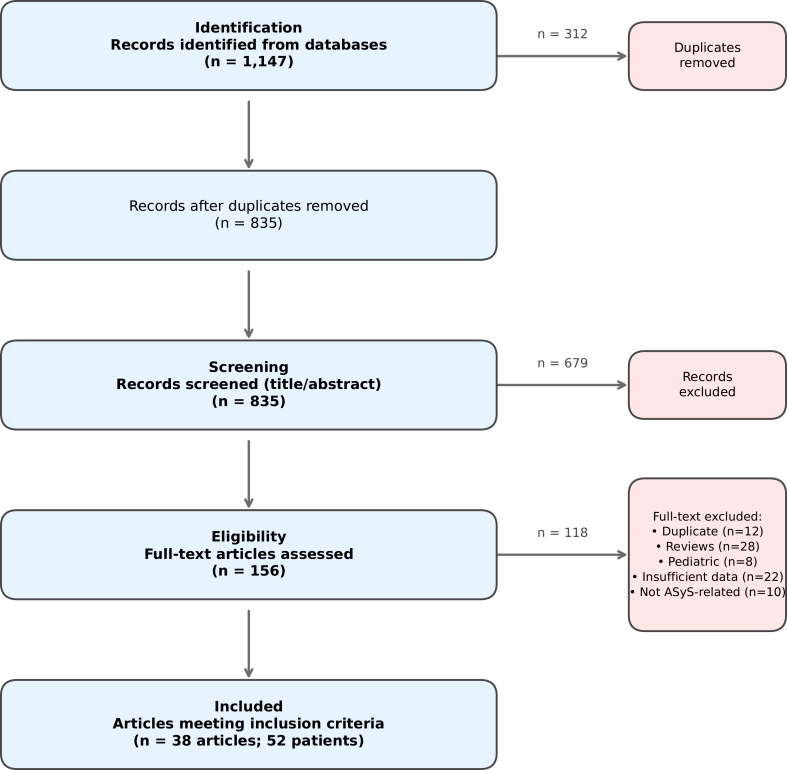
PRISMA 2020 flow diagram of study selection. The systematic search identified 1147 records from MEDLINE (via PubMed), Scopus, Web of Science, and Google Scholar. After removing 312 duplicates, 835 records underwent title and abstract screening. Of 156 full-text articles assessed for eligibility, 38 articles comprising 52 patients met inclusion criteria. Reasons for exclusion included duplicate publications (*n* = 12), reviews without original patient data (*n* = 28), pediatric cases (aged < 18 years; *n* = 8), insufficient clinical details (*n* = 22), and kidney involvement not attributable to ASyS (*n* = 10). ASyS, antisynthetase syndrome; PRISMA, Preferred Reporting Items for Systematic Reviews and Meta-Analyses.

The systematic search identified 1147 records. After removing 312 duplicates, 835 records underwent title and abstract screening, resulting in 156 full-text assessments. Thirty-eight articles met the inclusion criteria, comprising 52 patients (Figure 1). Of 118 full-text articles assessed for eligibility, 80 were excluded for the following reasons: duplicate publications (*n* = 12), reviews without original patient data (*n* = 28), pediatric cases (aged < 18 years; *n* = 8), insufficient clinical details (*n* = 22), and kidney involvement not attributable to ASyS (*n* = 10).

### Clinical Spectrum and Demographics

The median age at kidney presentation was 52 (range: 28–74) years, with female predominance (62%). Kidney disease presented concomitantly with or following ILD in 42 of 52 patients (81%). The median time from ASyS diagnosis to kidney involvement was 8 (interquartile range: 0–24) months.

Kidney manifestations ranged from asymptomatic proteinuria (*n* = 14, 27%) to rapidly progressive glomerulonephritis (*n* = 9, 17%). Mean peak serum creatinine was 3.4 ± 2.1 mg/dl (300 ± 186 μmol/l), with dialysis-dependent patients (*n* = 11, 21%) included in this calculation; when excluding dialysis patients, mean peak creatinine was 2.8 ± 1.6 mg/dl. Nephrotic-range proteinuria was present in 18 patients (35%), whereas 11 (21%) required dialysis at presentation (Table 1).

**Table 1:** Baseline demographic and clinical characteristics of patients with antisynthetase syndrome-associated kidney disease

Characteristics	*n* (%) median (range)
Total patients	52 (100)
Age at kidney presentation, yrs	52 (28–74)
Female sex	32 (62)
Time from ASyS diagnosis to kidney involvement, mo	8 (0–24)
Extrarenal manifestations	
Interstitial lung disease	42 (81)
Concurrent with kidney disease	28 (54)
Preceding kidney disease	14 (27)
Inflammatory myopathy	35 (67)
Arthritis/arthralgia	28 (54)
Mechanic’s hands	18 (35)
Raynaud phenomenon	12 (23)
Fever	15 (29)
Kidney presentation	
Asymptomatic proteinuria	14 (27)
Nephrotic-range proteinuria	18 (35)
Microscopic hematuria	22 (42)
Peak serum creatinine, mg/dl	3.4 ± 2.1
Dialysis requirement at presentation	11 (21)
Rapidly progressive glomerulonephritis	9 (17)
Autoantibody profile	
Anti-Jo1	24 (46)
Anti-PL-7	8 (15)
Anti-PL-12	7 (13)
Anti-EJ	4 (8)
Anti-OJ	1 (2)
Multiple ARS antibodies	3 (6)
Not specified	5 (10)
Biopsy performed	35 (67)

ARS, aminoacyl-tRNA synthetases; ASyS, antisynthetase syndrome.

### Histopathological Patterns

Kidney biopsy was performed in 35 patients (67%). The predominant histopathological patterns were membranous nephropathy (29%), pauci-immune crescentic glomerulonephritis (23%), acute tubular necrosis (17%), and tubulointerstitial nephritis (11%) (Table 2).

**Table 2:** Histopathological patterns in biopsy-proven antisynthetase syndrome-associated kidney disease (*n* = 35)

Histopathological pattern	*n* (%)	Key light microscopy features	lmmunofluorescence	Electron microscopy
Membranous nephropathy	10 (29)	Subepithelial deposits, GBM spike format	Granular IgG (10/10), C3 (8/)	Subepithelial deposits
PLA2R-positive	6 (60)	Spike formation, variable mesangial expansion	Granular IgG4 predominant	Subepithelial deposits with spikes
PLA2R-negative	4 (40)	similar light microscopy features	Granular IgG1/gG2, C3	Subepithelial deposits
Pauci-immune crescentic GN	8 (23)	Fibrinoid necrosis, cellular crescents (> 5q)	Negative or trace IgG (2/8)	No immune deposits
With ANCA positivity	5 (63)	Segmental necrosis capillaritis	Negative	Endothelial injury
ANCA negative	3 (37)	Similar necrotizing features	Negative	No deposits
Acute tubular necrosis	6 (17)	Tubular epithelial injury, loss of brush border	Negative	Tubular epithelial cell injury
Ischemic pattern	4 (67)	Patchy distribution, simplified epithelium	Negative	Mitochondrial swelling
Toxic pattern	2 (33)	Diffuse injury, nuclear loss	Negative	Cytoplasmic vacuolization
Tubulointerstitial nephritis	4 (11)	Interstitial inflammation, tubulitis	Negative or minimal lgG (1/4)	Interstitial edema
With granulomas	1 (25)	Noncaseating granulomas, eosinophils	Negative	Granulomatous inflammation
Without granulomas	3 (75)	Lymphoplasmacytic infitrate	Negative	Interstitial fibrosis (1/3)
IgA nephropathy	3 (9)	Mesangial proliferation, IgA deposits	Mesangial IgA (3/3), IgG (2/3)	Mesangial electron-dense depo
Minimal change disease	2 (6)	Normal light microscopy	Negative	Diffuse foot process effacement
Fibrillary GN	1 (3)	Mesangial expansion, Congo red-negative	lgG (1/1), C3 (1/1)	Fibrillary deposits (12–24 nm)
Thrombotic microangiopathy	1 (3)	Mesangiolysis, endothelial swelling	Fibrin (1/1), IgM (1/1)	Endothelial detachment

ANCA, antineutrophil cytoplasmic antibody; GBM, glomerular basement membrane; GN, glomerulonephritis; PLA2R, phospholipase A2 receptor.

Immunofluorescence studies in membranous cases revealed granular IgG deposition in 10 of 10 evaluated patients, with phospholipase A2 receptor (PLA2R) positivity on glomerular staining (not serum anti-PLA2R antibodies) in 6 cases (60%). This high prevalence of PLA2R positivity on tissue immunofluorescence in the context of systemic autoimmunity suggests possible secondary membranous nephropathy or coincident primary membranous nephropathy, as discussed later in this article.

### Autoantibody Associations and Clinical Phenotypes

Anti-Jo1 antibodies were present in 24 patients (46%) and were associated with milder kidney involvement (mean peak creatinine: 2.3 ± 1.1 mg/dl). Non-Jo1 antibodies (anti-PL-7, anti-PL-12, anti-EJ, and anti-OJ) were detected in 20 patients (38%) and demonstrated distinct clinical associations as follows:

Vasculitic phenotypes: Non-Jo1 antibodies were overrepresented in patients with pauci-immune crescentic glomerulonephritis or systemic vasculitis (70% vs. 25%; *P* = 0.02, unadjusted comparison)

Severity: Higher mean peak serum creatinine (4.7 ± 2.4 vs. 2.3 ± 1.1 mg/dl; *P* = 0.01, unadjusted comparison)

Recovery rates: Lower complete recovery with corticosteroid monotherapy (25% vs. 58%; *P* = 0.04, unadjusted comparison)

**Figure 2:**
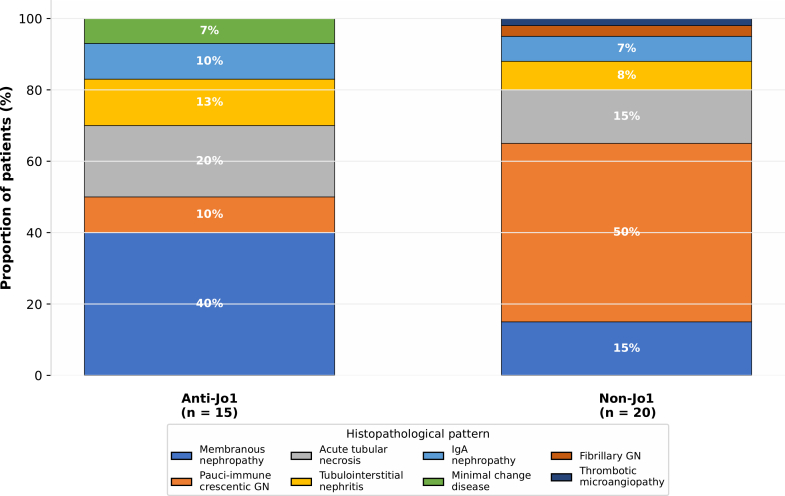
Distribution of kidney histopathological patterns according to autoantibody status (biopsy-proven cases only, *n* = 35). Membranous nephropathy was the most common pattern in anti-Jo1 positive patients (40%), whereas pauci-immune crescentic glomerulonephritis predominated in non-Jo1 antibody positive patients (50%). GN, glomerulonephritis.

In Figure 2, we illustrate the distribution of kidney histopathological patterns according to autoantibody status (biopsy-proven cases only, *n* = 35).

### Treatment Regimens and Kidney Outcomes

**Figure 3:**
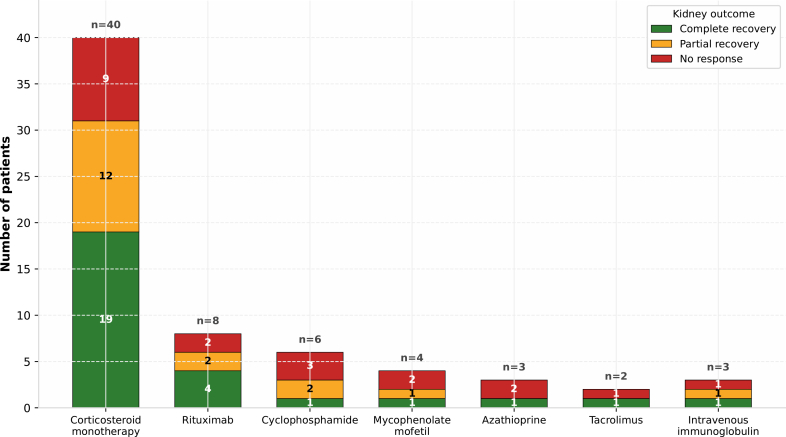
Kidney outcomes according to treatment modality. Complete recovery was defined as return to baseline or normalization of serum creatinine (< 1.2 mg/dl) plus resolution of proteinuria (< 0.3 g/24 h). Partial recovery required ≥ 50% reduction in proteinuria or ≥ 25% improvement in estimated glomerular filtration rate without meeting complete recovery criteria. No response indicates failure to achieve partial recovery criteria or progression to chronic kidney disease stages 4 and 5 or dialysis dependence. Numbers above bars indicate patient counts. This analysis includes all 52 patients; patients on dialysis at presentation who subsequently achieved dialysis independence were classified according to their final kidney function status.

First-line therapy consisted of high-dose corticosteroids (prednisone 0.5–1 mg/kg/d or pulse methylprednisolone) in 48 patients (92%). Second-line or adjunctive agents included cyclophosphamide (*n* = 6, 12%), mycophenolate mofetil (*n* = 4, 8%), azathioprine (*n* = 3, 6%), tacrolimus (*n* = 2, 4%), and i.v. Ig (*n* = 3, 6%). Rituximab (375 mg/m^2^/wk × 4 or 1 g × 2) was employed in 8 refractory patients. Outcomes according to treatment modality are summarized in Table 3 and Figure 3. Detailed patient-level treatment regimens and outcomes are provided in Supplementary Table S3.

**Table 3:** Treatment outcomes by modality in antisynthetase syndrome-associated kidney disease

Treatment modality	*n*	Complete recovery	Partial recovery	No response	Median FU (mo)	Keynotes
Corticosteroid monotherapy	40	19 (48)	12 (30)	9 (22)	18	First-line in mild/moderate cases
Rituximab	8	4 (50)	2 (25)	2 (25)	24	Refractory;4/5 pauci-immune GN achieved CR
Cyclophosphamide	6	1 (17)	2 (33)	3 (50)	12	Severe/vasculitic presentations
Mycophenolate mofetil	4	1 (25)	1 (25)	2 (50)	15	Maintenance or steroid-sparing
Azathioprine	3	1 (33)	0 (0)	2 (67)	10	Maintenance therapy
Tacrolimus	2	1 (50)	0 (0)	1 (50)	8	Refractory membranous nephropathy
Intravenous immunoglobulin	3	1 (33)	1 (33)	1 (33)	14	Adjunctive in severe cases

CR, complete recovery; eGFR, estimated glomerular filtration rate; FU, follow-up; GN, glomerulonephritis.

Some patients received multiple agents sequentially; counts reflect the primary or most intensive regimen. Complete recovery: return to baseline or normalization of serum creatinine (< 1.2 mg/dl) plus resolution of proteinuria (< 0.3 g/24 h). Partial recovery: ≥ 50% reduction in proteinuria or ≥ 25% improvement in eGFR without meeting complete recovery criteria. No response: failure to achieve partial recovery or progression to CKD stages 4-5 or dialysis dependence.

Rituximab (375 mg/m^2^/wk × 4 or 1 g × 2) was employed in 8 refractory patients, with 6 achieving partial or complete remission according to predefined criteria. Notably, 4 of 5 patients with pauci-immune crescentic glomerulonephritis treated with rituximab achieved complete recovery, compared with 1 of 3 treated with cyclophosphamide. However, these numbers are small and comparisons should be interpreted with extreme caution.

Among the 11 patients requiring dialysis at presentation, 6 (55%) achieved dialysis independence following immunosuppressive therapy (4 with corticosteroids alone, 2 with rituximab), whereas 3 (27%) remained dialysis-dependent at last follow-up and 2 (18%) died before renal recovery could be assessed.

At last follow-up (median [range]: 18 [3–84] months), 7 patients (13%) had progressed to CKD stages 4 and 5, and 3 (6%) remained dialysis dependent. Mortality was 8% (*n* = 4), predominantly attributable to progressive ILD and infectious complications.

### Sensitivity Analyses

Complete sensitivity analysis results are provided in Supplementary Table S2. Exclusion of 9 low-quality studies (Joanna Briggs Institute score < 6) did not substantially alter the distribution of histopathological patterns (membranous nephropathy 31% vs. 29% in full cohort) or the association between non-Jo1 antibodies and severe kidney injury (mean peak creatinine 4.5 vs. 4.7 mg/dl). Restricting to 35 biopsy-proven cases yielded similar findings for treatment responses (rituximab effectiveness 75% vs. 75%). Exclusion of 4 studies published before 2010 maintained the predominance of non-Jo1 antibodies in vasculitic phenotypes (68% vs. 70%).

When stratifying by diagnostic certainty, biopsy-proven cases (*n* = 35) showed similar autoantibody distributions and severity patterns compared with clinically defined cases (*n* = 17), though the latter group had lower rates of complete recovery (35% vs. 52%). This finding likely reflects more advanced disease at presentation in clinically defined cases, where clinicians may have foregone biopsy due to perceived futility or contraindications, rather than less severe disease. These analyses support the robustness of our main findings, though certainty remains very low because of inherent study limitations.

## Discussion

This systematic review of 52 patients with ASyS-associated kidney disease reveals several important preliminary findings that require validation in prospective studies. First, kidney involvement, although historically considered rare, represents a clinically significant complication based on cumulative case reports; however, the true incidence remains unknown because of ascertainment bias toward severe or atypical presentations.^11^ Second, the histopathological spectrum is remarkably heterogeneous, encompassing immune complex–mediated, pauci-immune, and tubulointerstitial patterns, suggesting multiple distinct pathophysiological mechanisms. Third, non-Jo1 autoantibodies, particularly anti-PL-7 and anti-PL-12, identify a subgroup potentially at higher risk for severe vasculitic kidney disease and worse outcomes, though this association requires confirmation in larger cohorts with multivariable adjustment.

### Clinical Implications

Our preliminary findings support consideration of routine kidney surveillance in all patients with ASyS, particularly those with non-Jo1 antibodies. Urinalysis and serum creatinine monitoring every 3 to 6 months represents a low-cost, high-yield strategy given the potential for silent progression; though this recommendation is based on expert opinion given the very low certainty of evidence.^12^

Therapeutic decision-making should be antibody-guided and histology-informed, pending validation in clinical trials. For mild tubular or membranous lesions, corticosteroid monotherapy may suffice, with 48% achieving complete recovery in our cohort. However, for crescentic or vasculitic presentations, strongly associated with non-Jo1 serotypes, early B-cell depletion with rituximab should be considered. The 75% response rate with rituximab in refractory cases aligns with its established efficacy in refractory myositis and ILD,^13^ and compares favorably with historical cyclophosphamide-based regimens, though these comparisons are limited by small sample sizes and selection bias.

### Pathophysiological Insights

The predominance of membranous nephropathy and PLA2R positivity in a subset of patients raises important mechanistic questions. The 60% PLA2R positivity rate observed in our cohort exceeds the expected prevalence of PLA2R-associated primary membranous nephropathy in the general population, suggesting several possibilities as follows: (i) molecular mimicry between aminoacyl-tRNA synthetase antigens and podocyte proteins may trigger PLA2R-specific autoimmunity as part of the ASyS spectrum; (ii) ASyS-related systemic inflammation may unmask or exacerbate subclinical primary membranous nephropathy; or (iii) these cases represent coincident primary membranous nephropathy in patients with ASyS, given that both conditions involve immune dysregulation.^6^ The temporal relationship between ASyS diagnosis and membranous nephropathy onset (concurrent or subsequent in most cases) favors the first 2 hypotheses, though definitive distinction requires prospective studies with serial antibody monitoring and genetic risk assessment.

Notably, we were unable to assess whether PLA2R positivity correlated with treatment response or kidney prognosis because of limited follow-up data. Future studies should specifically examine whether PLA2R serostatus predicts outcomes in ASyS-associated membranous nephropathy, analogous to primary membranous nephropathy.

Conversely, the association of non-Jo1 antibodies with pauci-immune crescentic disease implies distinct immunopathogenic pathways, possibly involving neutrophil extracellular trap formation and complement activation, analogous to antineutrophil cytoplasmic antibody–associated vasculitis.^14^ The frequent temporal relationship between ILD onset and kidney involvement (81% concurrent or subsequent) supports the hypothesis of shared epithelial or endothelial targeting in lung and kidney.^15^

### Methodological Considerations and Limitations

This review is constrained by inherent limitations of the source data that must be carefully considered when interpreting our findings. The retrospective, case-report nature of the included studies introduces substantial publication bias toward severe or atypical presentations; mild or subclinical kidney involvement is likely underrepresented. The small sample size (*n* = 52) precluded multivariable regression analysis, and all statistical comparisons should be considered exploratory and hypothesis-generating only.

The inclusion of both biopsy-proven and clinically defined kidney involvement, though enhancing sample size, introduces heterogeneity. Our sensitivity analyses suggest consistent patterns across both groups; however, clinically defined cases may include misclassified diagnoses or less severe disease. The lack of standardized outcome definitions across studies precluded meta-analysis. The Grading of Recommendations Assessment, Development and Evaluation assessment indicated very low certainty of evidence for all outcomes. Given that anti-Jo1 and non-Jo1 groups differed in age, time to kidney involvement, and other baseline characteristics, the observed differences are likely attributable to confounding rather than true group effects. These unadjusted comparisons should not be interpreted as establishing causal associations.

In addition, the evolving classification criteria for ASyS and variable antibody testing methodologies (enzyme-linked immunosorbent assay, immunoblot, or immunoprecipitation) across studies may have led to misclassification. Different detection methods vary in sensitivity and specificity, potentially affecting antibody subtype assignment. The median 18-month follow-up may be insufficient to capture long-term CKD progression, particularly in membranous nephropathy, where late relapses are well-documented.

### Future Directions

Prospective multinational cohorts are essential to define true incidence, validate risk stratification tools, and establish evidence-based treatment algorithms. We propose the establishment of an international ASyS kidney disease registry to facilitate standardized data collection and collaborative research. Biobanking of kidney tissue and serum samples would enable mechanistic studies of antibody-epitope interactions and PLA2R seropositivity patterns. Randomized trials comparing rituximab versus cyclophosphamide for severe kidney disease are warranted, though recruitment challenges in this rare condition will necessitate collaborative networks. Future studies should prioritize multivariable modeling using pooled multicenter data to adjust for confounders and validate the exploratory associations identified in this review.

### Conclusion

Kidney involvement in ASyS is heterogeneous and potentially clinically consequential. Non-Jo1 serotype and delayed immunosuppression emerge as potential predictors of adverse kidney outcomes, pending validation. Implementation of routine kidney surveillance and early antibody-guided immunosuppression, particularly B-cell depletion for aggressive presentations, may improve kidney survival, though these preliminary findings require confirmation in prospective studies. These findings underscore the urgent need for collaborative research to transform the current empiric approach into precision kidney care for this rare autoimmune condition.

## Disclosure

All the authors declared no competing interests.

## Supplementary Material

Supplementary File (PDF) **Supplementary Methods: Detailed Data Extraction Form. Supplementary References: excluded studies with reasons. Figure S1.** Risk of bias assessment. **Figure S2.** GRADE evidence profile. **Table S1.** Complete search strategy. **Table S2.** Sensitivity analysis results. **Table S3.** Detailed treatment regimens and outcomes.
