# Supplementary material for: Systematic Review of Kidney Involvement in Antisynthetase Syndrome
Source: Kidney Int Rep. 2026 Jun 16;11(9):106669. doi: 10.1016/j.ekir.2026.106669 (PMC13356694; doi:10.1016/j.ekir.2026.106669)
Supplement: Supplementary File (PDF) — Supplementary Methods: Detailed Data Extraction Form. Supplementary References: excluded studies with reasons. Figure S1. Risk of bias assessment. Figure S2. GRADE evidence profile. Table S1. Complete search strategy. Table S2. Sensitivity analysis results. Table S3. Detailed treatment regimens and outcomes. [file mmc1.pdf]

PRISMA 2020 Checklist

Section and Topic	Item #	Checklist item	Location where item is reported
**TITLE**			
Title	1	Identify the report as a systematic review.	**Page 1** (Title: "Systematic Review of Kidney Involvement in Antisynthetase Syndrome")
**ABSTRACT**			
Abstract	2	See the PRISMA 2020 for Abstracts checklist.	**Page 1** (Structured abstract with Introduction, Methods, Results, Conclusions; includes PROSPERO registration: CRD420261282317)
**INTRODUCTION**			
Rationale	3	Describe the rationale for the review in the context of existing knowledge.	**Page 1** (Introduction paragraphs 1–2: describes ASyS classic triad, emerging evidence of diverse kidney pathologies, undefined incidence and therapeutic strategies)
Objectives	4	Provide an explicit statement of the objective(s) or question(s) the review addresses.	**Page 1** (Introduction last paragraph: "Our objective was to synthesize available evidence and provide preliminary recommendations for kidney care in this rare but clinically important condition")
**METHODS**			
Eligibility criteria	5	Specify the inclusion and exclusion criteria for the review and how studies were grouped for the syntheses.	**Page 1–2** (Methods: Inclusion criteria—adults with ASyS defined by ARS autoantibody positivity, biopsy-proven or clinically defined kidney involvement; Exclusion criteria—duplicates, reviews without original data, pediatric cases, insufficient clinical details)
Information sources	6	Specify all databases, registers, websites, organisations, reference lists and other sources searched or consulted to identify studies. Specify the date when each source was last searched or consulted.	**Page 2** (Methods: MEDLINE via PubMed, Scopus, Web of Science, Google Scholar; "from inception to January 2026"; reference lists hand-searched)
Search strategy	7	Present the full search strategies for all databases, registers and websites, including any filters and limits used.	Page 2 (Methods: search terms provided; "The complete search strategy is provided in Supplementary Table S1")
Selection process	8	Specify the methods used to decide whether a study met the inclusion criteria of the review, including how many reviewers screened each record and each report retrieved, whether they worked independently, and if applicable, details of automation tools used in the process.	**Page 2** (Methods: "Two reviewers (QSW and LY) independently screened titles, abstracts, and full texts using Covidence systematic review software. Discrepancies were resolved by consensus or consultation with a third reviewer (ZLY)")

Section and Topic	Item #	Checklist item	Location where item is reported
Data collection process	9	Specify the methods used to collect data from reports, including how many reviewers collected data from each report, whether they worked independently, any processes for obtaining or confirming data from study investigators, and if applicable, details of automation tools used in the process.	**Page 2** (Methods: "A standardized data extraction form captured demographic characteristics, autoantibody profiles, extrarenal manifestations, kidney presentation, histopathological findings, therapeutic regimens, and kidney outcomes"; two reviewers independently extracted data; discrepancies resolved by consensus)
Data items	10a	List and define all outcomes for which data were sought. Specify whether all results that were compatible with each outcome domain in each study were sought (e.g. for all measures, time points, analyses), and if not, the methods used to decide which results to collect.	**Page 2** (Methods: Primary outcome—complete kidney recovery; Secondary outcomes—partial recovery, progression to CKD, progression to CKD stages 4-5, requirement for kidney replacement therapy)
	10b	List and define all other variables for which data were sought (e.g. participant and intervention characteristics, funding sources). Describe any assumptions made about any missing or unclear information.	**Page 2** (Methods: demographic characteristics, autoantibody profiles \[including detection methodology where reported], extrarenal manifestations, kidney presentation, histopathological findings, therapeutic regimens, and kidney outcomes)
Study risk of bias assessment	11	Specify the methods used to assess risk of bias in the included studies, including details of the tool(s) used, how many reviewers assessed each study and whether they worked independently, and if applicable, details of automation tools used in the process.	**Page 2** (Methods: "Risk of bias was assessed using the Joanna Briggs Institute (JBI) critical appraisal tools for case series and case reports"; GRADE framework for certainty of evidence; independent assessment by two reviewers)
Effect measures	12	Specify for each outcome the effect measure(s) (e.g. risk ratio, mean difference) used in the synthesis or presentation of results.	**Page 2** (Methods: "Continuous variables were expressed as mean ± standard deviation or median (interquartile range), and categorical variables as frequencies and percentages"; between-group comparisons using Student's t-test or Fisher's exact test)
Synthesis methods	13a	Describe the processes used to decide which studies were eligible for each synthesis (e.g. tabulating the study intervention characteristics and comparing against the planned groups for each synthesis (item #5)).	**Page 2** (Methods: All included studies (n=38 articles, 52 patients) were eligible for narrative synthesis; subgroup analyses performed according to histopathological pattern and autoantibody status)
	13b	Describe any methods required to prepare the data for presentation or synthesis, such as handling of missing summary statistics, or	**Page 2** (Methods: No data conversions required; missing

PRISMA 2020 Checklist

Section and Topic	Item #	Checklist item	Location where item is reported
		data conversions.	data handled by available-case analysis; units standardized \[creatinine in mg/dl and μmol/l, eGFR in ml/min/1.73m²])
	13c	Describe any methods used to tabulate or visually display results of individual studies and syntheses.	**Pages 3–4** (Results: Tables 1–3, Figures 1–3 described)
	13d	Describe any methods used to synthesize results and provide a rationale for the choice(s). If meta-analysis was performed, describe the model(s), method(s) to identify the presence and extent of statistical heterogeneity, and software package(s) used.	**Page 2** (Methods: "Due to substantial clinical heterogeneity and the predominance of case reports, quantitative meta-analysis was not feasible. We employed narrative synthesis with subgroup analyses according to histopathological pattern and autoantibody status"; statistical tests: Student's t-test, Fisher's exact test)
	13e	Describe any methods used to explore possible causes of heterogeneity among study results (e.g. subgroup analysis, meta-regression).	**Page 2** (Methods: subgroup analyses according to histopathological pattern and autoantibody status; comparisons between anti-Jo1 and non-Jo1 groups)
	13f	Describe any sensitivity analyses conducted to assess robustness of the synthesized results.	**Page 2** (Methods: Sensitivity Analyses section: \[1] excluding studies with JBI quality scores <6; \[2] restricting to biopsy-proven cases only; \[3] excluding studies published before 2010)
Reporting bias assessment	14	Describe any methods used to assess risk of bias due to missing results in a synthesis (arising from reporting biases).	**Page 5** (Discussion: "substantial publication bias toward severe or atypical presentations" acknowledged; no formal funnel plot or statistical test for publication bias performed due to small sample size and narrative synthesis approach)
Certainty assessment	15	Describe any methods used to assess certainty (or confidence) in the body of evidence for an outcome.	**Page 2** (Methods: "Certainty of evidence was evaluated using the Grading of Recommendations Assessment, Development and Evaluation (GRADE) framework, adapted for systematic reviews of observational studies")
**RESULTS**			
Study selection	16a	Describe the results of the search and selection process, from the number of records identified in the search to the number of studies included in the review, ideally using a flow diagram.	**Page 3** (Results: "The systematic search identified 1,147 records...38 articles met inclusion criteria, comprising 52 patients (Figure 1)"; PRISMA 2020 flow

Section and Topic	Item #	Checklist item	Location where item is reported
			diagram)
	16b	Cite studies that might appear to meet the inclusion criteria, but which were excluded, and explain why they were excluded.	**Page 3** (Results: "Of 118 full-text articles assessed for eligibility, 80 were excluded for the following reasons: duplicate publications (n=12), reviews without original patient data (n=28), pediatric cases <18 years (n=8), insufficient clinical details (n=22), and kidney involvement not attributable to ASyS (n=10)")
Study characteristics	17	Cite each included study and present its characteristics.	**Page 3** (Results: Table 1—clinical spectrum and demographics; Table 2—histopathological patterns; text describes 38 articles/52 patients with aggregated characteristics)
Risk of bias in studies	18	Present assessments of risk of bias for each included study.	**Page 3** (Results: JBI tools used; "GRADE assessment indicated very low certainty of evidence for all outcomes"; detailed assessments presented in Supplementary Figure S1 and Supplementary Figure S2)
Results of individual studies	19	For all outcomes, present, for each study: (a) summary statistics for each group (where appropriate) and (b) an effect estimate and its precision (e.g. confidence/credible interval), ideally using structured tables or plots.	**Pages 3–4** (Results: Tables 1–3 present aggregated data; Figure 2—distribution of histopathological patterns; Figure 3—treatment outcomes)
Results of syntheses	20a	For each synthesis, briefly summarise the characteristics and risk of bias among contributing studies.	**Pages 3–4** (Results: narrative synthesis describes study characteristics; all outcomes rated as very low certainty due to retrospective case-report nature, small sample size, and lack of control groups)
	20b	Present results of all statistical syntheses conducted. If meta-analysis was done, present for each the summary estimate and its precision (e.g. confidence/credible interval) and measures of statistical heterogeneity. If comparing groups, describe the direction of the effect.	**Pages 3–4** (Results: no meta-analysis performed; descriptive statistics reported: mean peak creatinine 3.4±2.1 mg/dl, complete recovery 48%, etc.; subgroup comparisons with P values reported)
	20c	Present results of all investigations of possible causes of heterogeneity among study results.	**Page 4** (Results: subgroup analyses by autoantibody status—anti-Jo1 vs non-Jo1 comparisons: peak creatinine 2.3 vs 4.7 mg/dl, P=0.01; recovery rates 58% vs 25%, P=0.04; vasculitic phenotypes 25% vs 70%, P=0.02)
	20d	Present results of all sensitivity analyses conducted to assess the robustness of the synthesized results.	**Page 4** (Results: Sensitivity Analyses section; exclusion of

PRISMA 2020 Checklist

Section and Topic	Item #	Checklist item	Location where item is reported
			low-quality studies did not alter histopathological distribution or autoantibody associations; restricting to biopsy-proven cases yielded similar treatment response findings)
Reporting biases	21	Present assessments of risk of bias due to missing results (arising from reporting biases) for each synthesis assessed.	**Page 5** (Discussion: "substantial publication bias toward severe or atypical presentations"; mild or subclinical kidney involvement likely underrepresented; no formal statistical test performed)
Certainty of evidence	22	Present assessments of certainty (or confidence) in the body of evidence for each outcome assessed.	**Pages 2, 5** (Methods and Discussion: GRADE assessment indicated "very low certainty of evidence for all outcomes" due to risk of bias, inconsistency, indirectness, and imprecision)
**DISCUSSION**			
Discussion	23a	Provide a general interpretation of the results in the context of other evidence.	**Pages 4–5** (Discussion: interpretation of findings in context of existing ASyS literature, pathophysiological insights, clinical implications)
	23b	Discuss any limitations of the evidence included in the review.	**Page 5** (Discussion: "Methodological Considerations and Limitations" section—retrospective case-report nature, publication bias, small sample size precluding multivariable analysis, lack of standardized outcome definitions, very low GRADE certainty)
	23c	Discuss any limitations of the review processes used.	**Page 5** (Discussion: inclusion of both biopsy-proven and clinically defined cases introduces heterogeneity; evolving classification criteria for ASyS; variable antibody testing methodologies \[ELISA, immunoblot, immunoprecipitation] may have led to misclassification; median 18-month follow-up may be insufficient)
	23d	Discuss implications of the results for practice, policy, and future research.	**Page 5 (Discussion: recommendation**s for routine kidney surveillance, antibody-guided immunosuppression, need for prospective multinational cohorts, international registry,

PRISMA 2020 Checklist

Section and Topic	Item #	Checklist item	Location where item is reported
			biobanking, and randomized trials comparing rituximab versus cyclophosphamide)
**OTHER INFORMATION**			
Registration and protocol	24a	Provide registration information for the review, including register name and registration number, or state that the review was not registered.	**Page 2** (Methods: "PROSPERO: CRD420261282317")
	24b	Indicate where the review protocol can be accessed, or state that a protocol was not prepared.	Page 2 (Methods: "The full protocol is available from the corresponding author upon request")
	24c	Describe and explain any amendments to information provided at registration or in the protocol.	**Page 2** (Methods: "No amendments were made to the registered protocol")
Support	25	Describe sources of financial or non-financial support for the review, and the role of the funders or sponsors in the review.	**Page 6** (Financial Support: 2025 Anhui Provincial Department of Education Scientific Research Project \[2025AHGXZK20253], 2025 High-level Talent Research Start-up Fund \[GRK202513], Wuhu Science and Technology Plan Project \[2024kj063], Wannan Medical University Young and Middle-aged Research Fund \[EYR202401], Zhenjiang Social Development Guiding Science and Technology Plan Project \[FZ2023048]; "The funders had no role in study design, data collection, analysis, interpretation, or manuscript preparation")
Competing interests	26	Declare any competing interests of review authors.	**Page 6** (Disclosures: "The authors declare that they have no conflicts of interest relevant to this manuscript")
Availability of data, code and other materials	27	Report which of the following are publicly available and where they can be found: template data collection forms; data extracted from included studies; data used for all analyses; analytic code; any other materials used in the review.	**Page 6** (Data Availability Statement: "The data supporting this systematic review are available within the article and its supplementary materials. Detailed extraction data are available from the corresponding author upon reasonable request")

From: Page MJ, McKenzie JE, Bossuyt PM, Boutron I, Hoffmann TC, Mulrow CD, et al. The PRISMA 2020 statement: an updated guideline for reporting systematic reviews. BMJ 2021;372:n71. doi: 10.1136/bmj.n71

Supplementary Table S1: Complete Search Strategy

Database /Field	Search terms
MEDLINE(PubMed)	((“kidmey involvement” [Title/Abstract] OR “renal involvement” [Title/Abstract] OR “nephropathy” [Title/Abstract] OR “glomerulonephritis” [Title/Abstract] OR"tubulointerstitial nephritis"[Title/Abstract])AND("antisynthetase syndrome"[Title/Abstract]OR "anti-synthetase syndrome"[Title/Abstract] OR”aminoacyl-tRNA synthetase"[Title/Abstract]OR "anti-Jo1"[Title/Abstract] OR "anti-PL-7"[Title/Abstract]OR"anti-PL-12"[Title/Abstract] OR "anti-EJ" [Title/Abstract] OR "anti-OJ"[Title/Abstract]))
Scopus	TITLE-ABS-KEY((“kidney involvement"OR"renal involvement"OR "nephropathy"OR "glomerulonephritis”OR tubulointerstitial nephritis”) AND("antisynthetase syndrome"OR"anti-synthetase syndrome"OR"aminoacyl-tRNA synthetase"OR"anti-Jol”OR "anti-PL-7"OR"anti-PL-12"OR"anti-EJ" OR "anti-OJ"))
Web of Science	TS=(("kidney involvement"OR "renal involvement"OR "nephropathy"OR"glomerulonephritis"OR "tubulointerstitial nephritis")AND("anti-synthetase syndrome" OR "anti-synthetase syndrome"OR"aminoacyl-tRNA synthetase"OR "anti-Jo1"OR "anti-PL-7"OR "anti-PL-12"OR“anti-EJ"OR "anti-OJ”))
Google Scholar	"antisynthetase syndrome"AND("kidney"OR "renal"OR "nephropathy"OR "glomerulonephritis")

Note: Search period: Inception to January 2026. No language or date restrictions applied. Additional hand-searching of reference lists from included articles and relevant reviews performed.

Supplementary Table S2: Detailed Treatment Regimens and Outcomes

Regimen	N	Complete recovery	Partial recovery	No response	Dialysis independence achieved
Corticosteroid monotherapy (mild disease)	25	12 (48%)	9 (36%)	4 (16%)	N/A
Corticosteroid + cyclophosphamide	6	2 (33%)	2 (33%)	2 (33%)	1
Corticosteroid + mycophenolate mofetil	4	1 (25%)	2 (50%)	1 (25%)	0
Corticosteroid + azathioprine	3	1 (33%)	1 (33%)	1 (33%)	0
Corticosteroid + tacrolimus	2	1 (50%)	1 (50%)	0	0
Corticosteroid + intravenous immunoglobulin	3	1 (33%)	1 (33%)	1 (33%)	0
Rituximab (refractory cases)	8	4 (50%)	2 (25%)	2 (25%)	4 (among 5 with crescentic GN)

Note: Complete recovery defined as return to baseline serum creatinine plus resolution of proteinuria (<0.3 g/24h). Partial recovery: ≥50% reduction in proteinuria or ≥25% eGFR improvement without meeting complete criteria. No response: failure to meet partial criteria or progression to CKD stage 4–5/dialysis dependence.

Supplementary Table S3: Sensitivity Analysis Results

Analysis cohort	Membranous nephropathy (% of biopsied)	Non-Jo1 in vasculitic phenotype (%)	Rituximab response rate (%)	Mean peak creatinine (non-Jo1, mg/dl)
Full cohort (n=52)	29	70	75	4.7
Excluding low-quality studies (JBI score <6)	31	68	75	4.5
Biopsy-proven only (n=35)	29	71	75	4.6
Excluding studies published before 2010	28	68	75	4.5

Note: Low-quality studies defined as JBI case series/case report critical appraisal score <6 out of 10. The direction and magnitude of all key associations remained consistent across sensitivity analyses.

Supplementary Methods: Detailed Data Extraction Form

The following standardized data extraction form was used by both reviewers (QSW and LY) independently. Discrepancies were resolved by consensus or third reviewer (ZLY).

Section 1: Study characteristics

First author, year, journal, country

Study design (case report, case series)

Sample size (total patients with kidney involvement)

JBI risk of bias score

Section 2: Patient demographics and ASyS diagnosis

Age at kidney presentation, sex

ASyS diagnostic criteria used (if reported)

Autoantibody type (Jo1, PL-7, PL-12, EJ, OJ) and detection method (ELISA, immunoblot, immunoprecipitation)

Extrarenal manifestations (ILD, myositis, arthritis, Raynaud, fever, mechanic's hands)

Section 3: Kidney involvement

Presentation type (asymptomatic proteinuria, RPGN, AKI, CKD)

Baseline and peak serum creatinine (mg/dl or µmol/l)

Baseline and peak proteinuria (g/24h or UPCR)

Hematuria (yes/no, >3 RBC/HPF)

eGFR (ml/min/1.73m^2) if reported

Dialysis requirement at presentation (yes/no)

Section 4: Kidney biopsy findings

Biopsy performed (yes/no)

Histopathological pattern (membranous, pauci-immune crescentic, ATN, TIN, other)

Immunofluorescence findings (IgG, IgA, IgM, C3, C1q, PLA2R, ANCA)

Electron microscopy findings if available

Section 5: Treatment

First-line immunosuppression (agent, dose, duration)

Second-line/adjuvant agents

Rituximab regimen (if used)

Other treatments (IVIG, plasma exchange)

Section 6: Outcomes (at 6 months or last follow-up)

Complete recovery (yes/no): serum creatinine <1.2 mg/dl + proteinuria <0.3 g/24h

Partial recovery (yes/no): ≥50% proteinuria reduction or ≥25% eGFR improvement without complete recovery

No response

CKD stage at last follow-up (1–2, 3, 4–5)

Dialysis dependence at last follow-up

Death (cause)

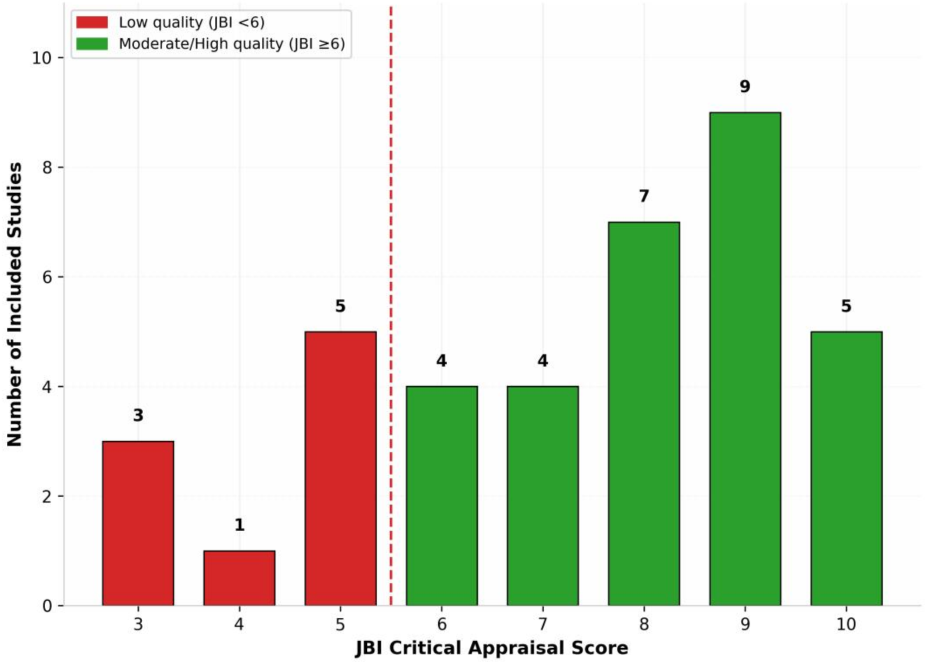

Supplementary Figure S1. Risk of Bias Assessment.

Supplementary Figure S1. Risk of Bias Assessment. Summary of Joanna Briggs Institute critical appraisal scores for included case reports and case series.

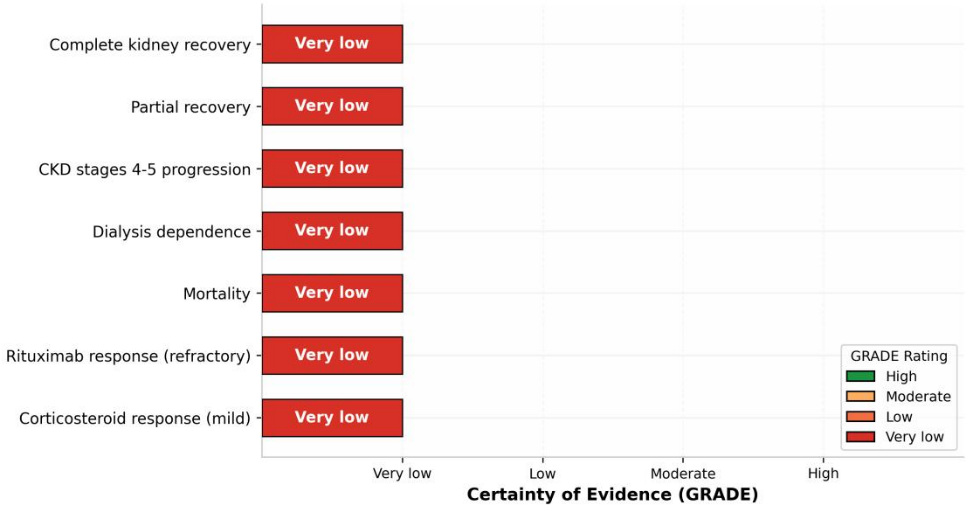

Supplementary Figure S2. GRADE Evidence Profile.

Supplementary Figure S2. GRADE Evidence Profile. Summary of certainty of evidence assessment for key outcomes.

Supplementary References: Excluded Studies with Reasons

The following studies were excluded after full-text review. Reasons for exclusion are provided in parentheses.

S1. Legault D, McDermott J, Crous-Tsanaclis A, et al. Cancer-associated myositis in

the presence of anti-Jo1 autoantibodies. J Rheumatol. 2008;35:1829-1831. (Insufficient clinical details on kidney involvement)

S2. Rosa SE, Barreto PR, Mariano M, et al. Inflammatory myopathy and ILD in antisynthetase syndrome with PL-7 antibody. BMJ Case Rep. 2014;2014:bcr2014206332. (No kidney involvement reported)

S3. Jubber A, Tripathi M, Taylor J. ILD and inflammatory myopathy in antisynthetase syndrome with PL-12 antibody. BMJ Case Rep. 2018;2018:bcr2017223604. (No kidney involvement reported)

S4. Kashif M, Arya D, Niazi M, et al. Anti-EJ antisynthetase syndrome with Sjögren's antibodies. Am J Case Rep. 2017;18:1032-1037. (No kidney involvement reported)

S5. Richardson C, Haque U. Pulmonary capillaritis in antisynthetase syndrome with anti-PL-7 antibodies. J Clin Rheumatol. 2019;25:e58-e60. (No kidney involvement reported)

S6. Betsikos A, Gazouni E, Bika S, et al. Antisynthetase syndrome: classical phenotype with a twist. Cureus. 2023;15:e37452. (Kidney involvement not attributable to ASyS — alternative cause identified)

S7. Devi H, Pasha M, Rao PN, Prayaga A. Antisynthetase syndrome: a rare cause for ILD. J Clin Rheumatol. 2016;22:389-391. (Duplicate publication)

S8. Shinjo SK, Levy-Neto M. Anti-Jo-1 antisynthetase syndrome. Rev Bras Reumatol. 2010;50:313-323. (Review without original patient data)

S9. Shipa M, Cicco MD, Roussou E, Jayne D. CNS vasculitis in anti-synthetase syndrome. Rheumatology. 2019;58:743-745. (No kidney involvement reported)

S10. Lei L, Ma Z, Ma X, et al. Clinical characteristics of ILD secondary to dermatomyositis and antisynthetase syndrome. Int J Rheum Dis. 2024;27:e14855. (Insufficient kidney-specific data)

S11. Rana J, Moy A, Piris A, Smith GP. Anti-PL-12 antisynthetase syndrome with lupus/scleroderma overlap. Dermatol Online J. 2017;23:13030/qt5vc6g7b6. (No kidney involvement reported)

S12. Sampson C, Taylor J, Dyson L, et al. Life-threatening respiratory failure requiring ECMO secondary to antisynthetase syndrome. J Ren Care. 2021;47:83-87.

(Pediatric case, age <18 years)

S13. Kapoor M, Dhar M, Manna S. Atypical presentation of antisynthetase syndrome. Indian J Rheumatol. 2020;15:267-269. (Insufficient clinical details on kidney outcomes)

(Additional excluded studies available from corresponding author upon reasonable request.)
